# Dietary diversity and associated factors among children (6–23 months) in Gedeo zone, Ethiopia: cross - sectional study

**DOI:** 10.1186/s13052-021-01181-7

**Published:** 2021-12-11

**Authors:** Wondwosen Molla, Dirshaye Argaw Adem, Ruth Tilahun, Seid Shumye, Robel Hussen Kabthymer, Daniel Kebede, Nebiyu Mengistu, Getnet Melaku Ayele, Dawit Getachew Assefa

**Affiliations:** 1 Department of Midwifery, Dilla University, Dilla, Ethiopia; 2 School of Public health, Dilla University, Dilla, Ethiopia; 3 Department of Psychiatry, Dilla University, Dilla, Ethiopia

**Keywords:** Dietary diversity, Minimum dietary diversity score, Ethiopia

## Abstract

**Introduction:**

Different foods and food groups are good sources for various macro- and micronutrients. Diversified diet play an important role in both physical and mental growth and development of children. However, meeting minimum standards of dietary diversity for children is a challenge in many developing countries including Ethiopia.

**Objective:**

**To assess** dietary diversity and associated factors among children (6–23 months) in Gedieo Zone, Ethiopia.

**Method:**

Community based cross-sectional study was carried out at Gedieo Zone, Ethiopia, from January to March 15, 2019. Multi-stage sampling technique was used to get a total of 665 children with the age of between 6 and 23 months from their kebeles. Data was collected by using face-to-face interview with structured questionnaire. Data was entered into Epidata version 3.1 and exported to Statistical Package for the Social Sciences (SPSS) version 23.0 for analyses. Variables having *p* < 0.25 at bivariate analysis were fitted to multivariable analysis. Multivariable logistic regression model was used at 95% confidence interval and with *P*-Value < 0.05. Bivariate.

**Result:**

A total of 665 children were participated with response rate of 96.2%. Only 199(29.9%) of children were met the minimum requirements for dietary diversity. Age of children [AOR 4.237(1.743–10.295))], Educational status [AOR 2.864(1.156–7.094)], Number of families [AOR 2.865(1.776–4.619))] and household wealth index [AOR4.390(2.300–8.380)] were significantly associated with Dietary Diversity of children.

**Conclusion:**

Only, one out of four children aged of 6–23 months attained the minimum dietary diversity score. Children from low socioeconomic status and mothers with no formal educational attainment need special attention to improve the practice of appropriate feeding of children.

## Introduction

Dietary diversity is the sum of food groups that is consumed by any individual within a period of 24 h [1], which has been documented as a valid and reliable indicator of dietary adequacy. Currently, available scientific evidence revealed that dietary diversity is strongly associated with dietary quality and nutrient adequacy. In fact, there is no single food which contains all required nutrients for optimal health [2].

Different foods and food groups are good sources for various macro and micronutrients to ensure nutrient adequacy [3]. Adequate nutrition is important to meet both known and as yet unknown needs for human health. For example, it is fundamental for physical and mental, growth and development of children, healthy living, associated with reduction of risk of mortality and morbidity for children and highly correlated with caloric and protein adequacy and consumption associated with improved nutritional status of children [4–6].

The first 2 years of life are a critical window to promote optimal child growth and development of the child. World Health Organization (WHO) recommends to initiate nutritionally adequate, safe, and appropriate complementary food at the age of sixth month [7]. After the age of sixth month, the energy and nutrient content of breast milk alone is not enough to meet the nutritional demand of the growing infant to prevent stunting among children. Therefore, providing sustainable diets rich in micronutrients and macronutrients is vital in the effort to combat malnutrition to children [8].

Globally, around 5.9 million under-five age children are dead annually, majority of them are in Africa, especially in sub-Saharan Africa including Ethiopia. Childhood malnutrition is the most pressing public health problem [9], predominantly in developing countries as it has primarily has been directly or indirectly for 45% which is 5.9 million deaths of under-five children globally, majority of this death occurs in Africa. In Ethiopia, more than 38.4% of children are stunted, 9.9% are wasted, and 23.6% are underweight indicating the persistence of both acute and chronic under nutrition according to the 2016 Ethiopian Demographic and Health Survey [10]. Greater than two-thirds of malnutrition-related child deaths are associated with inappropriate feeding practices during the first 2 years of life in such a way that infants and young children received inadequately nutritious diets and were poorly diversified [11]. In Sub-Saharan African Regions, suboptimal infant feeding practices, poor quality of complementary foods, micronutrient deficiencies, and frequent infections have mainly contributed to the high mortality among infants and young children [12].

Furthermore, malnourished children are at higher risk for childhood illness like diarrheal diseases and infections, impaired cognitive development, growth retardation, smaller adult stature, and a consequence of compromised educational achievement and low economic productivity which become impossible to reverse later in life [13–16]. Child under-nutrition is caused by numerous and multidimensional factors; however, lack of diversified or adequate food is the major factor for the problem [13, 17].

Inadequate or lack of dietary diversity is the major cause of malnutrition, it does not provide adequate calories and micronutrients for individuals [18]. Micronutrient deficiency is a risk to children and is referred to as hidden hunger [19]. It is caused by chronic deficiency of vitamins and minerals as a consequence of nutrient inadequacy [20].

Therefore, appropriate, safe, adequate and frequent child feeding practice is fundamental for optimal growth, better health and development of children. Available scientific evidence revealed that consumption of a diversified diet is important in the reduction of under-nutrition among infants and young children aged 6 to 23 months [21, 22] and also, World Health Organization (WHO) and Infant and Young Child Feeding (IYCF) recommend that both breastfed and non-breastfed children should consume four or more food groups among the seven food groups, which provide sufficient energy, protein, and micronutrients for infants and young children aged 6 to 23 months. These food groups are: grains roots and tubers, legumes and nuts, dairy products (milk, yogurt, cheese), flesh foods (meat, fish, poultry, and liver/organ meats), eggs, vitamin- A rich fruits and vegetables and other fruits and vegetables [7, 23].

Furthermore, because of the perceived importance of dietary diversity for health and nutrition, indicators of dietary diversity have become increasingly popular in recent years [24]. World Health Organization (WHO) uses dietary diversity as one of the key indicators to assess child feeding practices [7] i.e., individuals consuming more diverse diets are considered more likely to meet their nutrient needs [25]. Besides the national nutrition program of Ethiopia, the country declared at Seqota declaration which pays particular attention to the importance of nutrition in pregnancy and in the first years of a child’s life to stop the cycle of under nutrition [26].

However, meeting minimum standards of dietary diversity is a major challenge in many developing country settings including Ethiopia which is as low as 7% [27]. According to the recent study conducted in the world revealed that occupation of mothers, women’s empowerment, household food security, father’s educational status, exposure to media age of the child in months, age of the mother, socio-economic status number of under-five children in the household has a significant association with chilled dietary diversity [6, 28, 29].

The causes of childhood malnutrition are diverse, multidimensional, and interrelated. Even if people get enough to eat, good nutrition requires access to a sufficient supply of varied, safe and nutritious food to meet daily nutritional requirements. An essential element of food-based approach involves dietary diversification. However, food access may be affected by different conditions, like market conditions, cultural and religious practices. The aim of this study was to assess children dietary diversity and association factors. Which might be essential for program managers and stakeholders in identifying specific strategies to improve child nutrition. It plays a major role to reduce child mortality and morbidity. Furthermore, it provides baseline information to formulate program for accelerating malnutrition intervention activities and help governmental and nongovernmental organizations /NGO/ which work on food-based approaches to meet the micronutrient needs of mothers and children in the study area.

## Methods

### Study area and period

This study was conducted at Gedeo Zone, Ethiopia. Gedeo zone is located in Southern which is 360Km far from Addis Ababa, capital city of Ethiopia with the administrative center of Dilla town, Sidama in the South, Abaya in the North, H/Mariam in the East and Kericha in the West bounding the zone. Gedeo zone has six districts and two city administrations and has a total population of 1,086,768 (532,516(49%) males and 554, 225(51%) females, with an area of 1210.89 km^2^ [30]. And in the zone there are 6 districts and two town cities with 164 kebeles with 31 urban kebeles and 133 rural kebeles with a total of 276 health facilities from this one referral hospital, three district hospitals,38 health centers,146 health posts, five NGO clinics, 36 private clinics and 47 drug venders. The study was conducted from January1 to February 15, 2019 GC.

### Study design

Community based cross-sectional study was conducted.

### Source population

All children aged 6–23 months paired with their mothers who lived in Gedeo Zone.

### Study population

All children aged 6–23 months paired with their mothers from the selected kebeles.

### Eligibility criteria

#### Inclusion criteria

Mothers or caregivers of children aged 6–23 months who were permanent residents in Gedeo zone for the last 6 months were included in the study.

#### Exclusion criteria

Mothers or caregivers who have a health problem that can affect the interview process and households that had a special ceremony on the day prior to data collection were excluded from the study.

### Sample size determination

The sample size was determined by using single population proportion formula taking 0.05 margin of errors at 95% confidence level. Considering the fact that the proportion closer to 50% will give the largest sample size. It was used. n = (Zα/2)2p (1 − p)/d2, where

n = minimum sample size,

Z 1-α/2 = significance level at α =0.05 (standard normal variable at 95% confidence level = 1.96)

d = expected margin of error (5%)

P = proportion of children DD (50%)

Since, multi-stage sampling technique was used. Therefore, the sample size was multiplied by the design effect of 1.5 for possible non-response rate during the study, the final sample size was increased by 20% to: *n* = 691.

### Sampling technique

A multi-stage sampling technique was used. Initially, out of 6 districts of the Zone, three districts are selected by using simple random sampling techniques (lottery method). Namely, Yirgacheffie (31kebeles), Bule (29kebeles) and Dilla Town (9kebeles). From a total of 69 kebeles; 11, 10 and 3 kebeles are selected by using simple random sampling techniques from Yirgacheffie district, Wonago district and Bule district respectively. The final sample size was allocated proportionally for each kebeles based on the number of children. Finally, respondents were selected by using a simple random sampling technique.

### Study variable

#### Dependent variable

Dietary Diversity of children from the age of 6–23 months’

#### Independent variables

Socio-demographic and economic factors: Maternal (age, educational level, occupation, status in household), child age, birth order, breastfeeding status, starting time of complementary feeding, child sex, residence, household wealth, family size, chicken rearing, milking cow, vegetable gardening, Health care related factors: Antenatal care (ANC), postnatal care (PNC), Delivery site, follow-up programs for Growth Monitoring program (GMP), Vaccination, Dietary advice, Morbidity related factors: Child infection, Food refusal of children, Diet and food access related factors, Household food insecurity, Primary Source of food.

### Operational definition of terms

Minimum dietary diversity score (the number of food groups the child consumed during the 24-h preceding the survey) was used as a proxy for dietary diversity. It was calculated and divided into two categories of meeting the minimum dietary diversity or not (i.e., consumption of < 4 or ≥ 4 food groups) based on the WHO guidelines who fed > 4 groups of foods from the seven food groups in 24 h. The time period was considered as met the minimum dietary diversity of children [7].

Child DD - 24 h’ qualitative dietary recall data of the children were collected from the mothers who were responsible for feeding during the previous day of the study.

### Data collection methods

The data was collected by using face-to-face interviews with a structured questionnaire. The questionnaire was prepared in English then translated to Amharic and Gedieo offa languages, then back-translated to English by an independent translator for its consistency.

Data was collected by using 15 data collectors and 5 supervisors who had a diploma and above in the health profession. Three days training was given for data collectors and supervisors on the overall procedure of the study.

### Data analysis

Data were checked for completeness, edited, coded. The data was entered by using Epi data version 3.1 software then exported to SPSS version 23.0 statistical software for analysis. Descriptive statistics such as mean, median, frequency and percentage were used. Bivariate analysis was done and all explanatory variables with *P*-value less than 0.25 was regressed in to multivariable analysis. Multivariable analysis was employed to determine independent determinant factor among explanatory variables. Adjusted odds ratio (AOR), 95% confidence interval and P-value less than or equal to 0.05 was used to decide a statistically significant association with the outcome variable. Model fitness was assessed by using Hosmer and Lemeshow test. Multicollinearity was checked by using variance inflation factor (VIF) and tolerance test. The result of VIF was less than 2 while the tolerance test was greater than 0.1, which was within the normal limit. The finding of this study presented in the form of text, charts and tables.

### Data quality

To keep the data quality, standard questionnaire was adapted. The data collectors and supervisors were trained for 02 days on the aims of the research, content of the questionnaire and how to conduct the interview to increase their performance in the activities. Data was collected on all days of the week since people may eat differently on different days of the week. All interviews were conducted at the residences of the study participants. Vacant or closed houses during the day of visit was revisited two times to maintain the required sample size.

The Collected data was checked every day by the supervisors and principal investigator for its completeness and consistency. All questionnaires were kept under lock and key for security and confidentiality of obtained information.

### Dissemination of the result

The finding of the study is presented to College of Medicine and Health Science, Dilla University. The findings of the study will be distributed to all health facility staffs and other organizations working on nutrition and maternity and child health. The findings will also be presented in different seminars, meetings and workshops and publication in scientific journal will be considered to enable for wider access.

## Result

### Socio demographic characteristics of the mother

A total of 665 mothers of children from the age of 6 to 23 months were participated in this study, making the response rate of 96.2%. All participants, 665 (100%) of them were biological mothers. Majority 315(47.4%) of the respondents were in the age range of 25–29 years. Most of them 368(55.3%) were Gedeo in ethnicity, 304 (45.7%) were protestant. Furthermore, majority of 499(75%) of respondents were Married, 412(62%) were housewives and majority 375(56.4%) of them were unable to read and write. As indicated Table 1.

**Table 1:** Socio-demographic characteristics of respondents in gedeo zone, SNNPR, Ethiopia 2020

Variable	Category	Frequency	Percent
Age of mothers	< 20	107	16.1
	20–24	133	20.0
	**25–29**	**315**	**47.4**
	> = 30	110	16.5
Marital status	**Married**	**499**	**75.0**
	Single	59	8.9
	Divorced	65	9.8
	Widowed	42	6.3
Ethnicity	**Gedeo**	**368**	**55.3**
	Oromo	133	20.0
	Amhara	85	12.8
	Other*	79	11.9
Religion	Orthodox	219	32.9
	**Protestant**	**304**	**45.7**
	Muslim	107	16.1
	other	35	5.3
Educational status	**Cannot read and write**	**375**	**56.4**
	read and write	206	31.0
	Formal education	84	12.6
Occupation	**Housewife**	**412**	**62.0**
	Student	27	4.1
	Governmental employer	30	4.5
	Daily laborer	108	16.2
	Merchant	88	13.2
Family size	**< 4**	**351**	**52.8**
	> = 4	314	62.1
Primary source of obtaining food	Farming	223	33.5
	**Buying from market**	**422**	**66.5**
Head of house hold (mothers)	Yes	272	40.9
	**No**	**393**	**59.1**
Antenatal clinic visits	**Yes**	**621**	**93.4**
	No	44	6.6
Postnatal care	**Yes**	**590**	**88.7**
	No	75	11.3
Place of delivery	**Institution**	**633**	**95.2**
	Home	32	4.8
Birth order	First order	147	22.1
	2–3	204	30.7
	**> 3**	**314**	**47.2**
Household wealth	Poor	220	33.1
	**middle**	**223**	**33.5**
	Rich	222	33.4

Others like; Sidama, Tigre, Wolayita

### Children characteristics

From the total of 665 of children (6 to 23 months), the mean age of children was 15.1 months with ±5.16SD. Majority 289(43.5%) of them were found in age between 12 to 18 months followed by 19–23 months which is 186(28.2%). Almost half 321(48.3%) of the children who took part in the study were female. Majority, 651(97.9%) of children were breastfed at least once time in their life, and 547(82.3%) of children were breastfeeding at the time of data collection.

From the total 665 mothers of children, majority 381(57.3%) of mothers introduced CF after the sixth month age of children followed by 215(32.3%) of them started complimentary feeding (CF) during the infant’s sixth month of age while the rest 69(10.4%) before the sixth month age of children. Majority 626(94.1%) of them were vaccinated, 531(79.8%) had follow-up programs for Growth Monitoring program (GMP) at health facility. As indicated Table 2.

**Table 2:** Children characteristic at Gedeo zone, SNNPR, Ethiopia 2020

Variable	Category	Frequency	Percent
Age of children	6–8	82	12.4
	9–11	108	16.6
	**12–18**	**289**	**43.5**
	19–23	186	28.2
Sex of children	Female	321	48.3
	***Male***	***344***	***51.7***
Currently breast feeding	***Yes***	***547***	***82.3***
	***no***	***118***	***17.7***
Starting time of complementary feeding	***Before 6 months***	***69***	***10.4***
	***At 6 months***	***215***	***32.3***
	***After 6 months***	***381***	***57.3***
Infection or fever	***Yes***	***197***	***29.6***
	No	468	70.4
Food refusal	***Yes***	***271***	***40.8***
	No	394	59.2
GMP	***Yes***	*531*	*79.8*
	No	**134**	**20.2**
Vaccinated	***Yes***	**626**	**94.1**
	No	39	5.9

### Dietary diversity of children

**Fig. 1:**
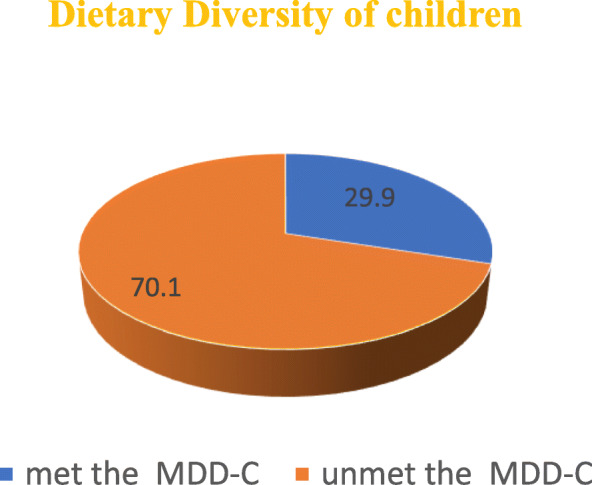
dietary diversity scores of children at Gedeo zone, SNNPR, Ethiopia 2020

The overall children who met the requirement of minimum dietary diversity per 24 h were 29.9% (199), see Fig. 1.

### Consumption of food groups

**Fig. 2:**
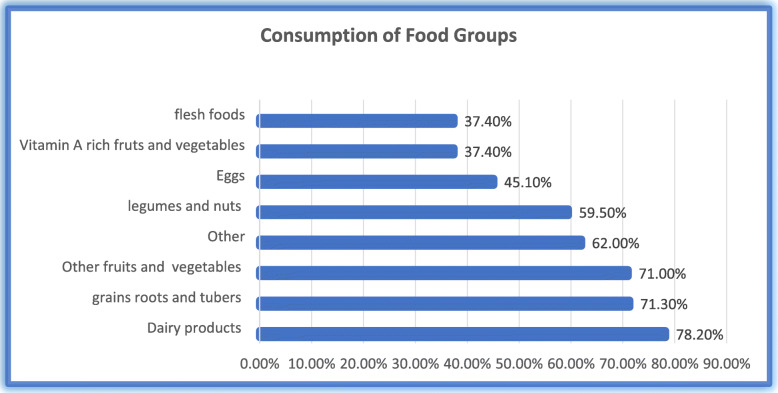
Consumption of food groups by children in Gedieo Zone, SNNPR, Ethiopia, 2020

Consumption of foods by children based on seven food groups, majority of them 520(78.2%) were consumed dairy products per 24 h before data collection which is the predominant one followed by grains, white roots and tubers (71.3%), other fruit and vegetables (71%), other like foods made with any oil, fats, butter and or sugary foods, such as chocolates, sweets, candies, or biscuits(62%), Legumes and nuts(59.5%), egg(45.1%) while the least consumed food groups were vitamin A rich fruits and vegetables and flesh foods which is 249 (37.4%) for both as see Fig. 2.

### Determinants of dietary diversity among children

In the bivariate logistic regression analysis, age of mother, mother’s education status, number of family/children, head of the household, primary source of food, meal frequency, presence of infection or fever, household wealth, sex of children and age of children were the candidate variables for multi-variables analysis. Whereas in multivariable logistic regression analysis, household wealth index [AOR4.390(2.300–8.380)], Educational status [AOR 2.864(1.156–7.094)], Number of families [AOR 2.865(1.776–4.619))] and age of children, [AOR 4.237(1.743–10.295))] were significantly associated with Dietary Diversity of children as see Table 3.

**Table 3:** Bivariate and multivariable analysis on factors associated with dietary diversity among children at Gedeo zone, SNNPR, Ethiopia 2020

Variables	Dietary diversity		COR (95% CI)	AOR (95% CI)	*P* value
	<MDDS	>MDDS			
Educational status **(mother)**					
Cannot read and write	310	65	1	1	
read and write	139	67	2.298(1.465–3.382) **	5.173(2.132–12.552)	0.04
***Formal education***	**33**	**51**	7.370(5.329–15.171) *	**2.864(1.156–7.094)**	**0.00**
Age of respondent **(mother)**					
< 20	71	36	2.773(2.068–9.619) **		
*20–24*	75	58	4.2306(3.709–16.125) *		
25–29	227	88	2.1208(1.209–4.969) **		
> =30	93	17	1		
**Number of family**					
**< 4**	**114**	**84**	2.025(1.437–2.854) *	**2.865 (1.776-4.619)**	**0.00**
> =4	322	115	1	1	
**Primary source of food**					
**Farming**	**291**	**151**	1.892(1.300–2.753) **		
Buying from market	175	48	1		
**Infection for children**					
Yes	171	26	1		
**No**	**295**	**173**	3.857(2.451–6.069) *		
**Meal frequency**					
< =2 times	221	58	1		
**> 2 times**	**245**	**141**	2.1929(1.414–2.882) **		
**Sex of children**					
**Male**	**199**	**143**	2.7764(1.567–5.086) **		
female	255	66	1		
**Age of children**					
6–8	111	21	1	1	
**9–11**	**52**	**56**	5.6923(1.211–5.288) *	**4.237(1.743–10.295)**	**0.00**
12–18	171	68	2.1019(1.409–3.283) **	3.471(1.547–7.786)	0.02
19–23	133	53	2.1063(1.567–3.086) **	3.957(1.726–9.076)	0.03
Household wealth index					
***Rich***	**123**	**99**	4.4032(2.276–9.066) *	**10.430(5.288–20.575)**	**0.00**
middle	157	66	2.2997(1.483–5.323) **	4.390(2.300–8.380)	0.04
Poor	186	34	1	1	

## Discussion

This study has investigated the dietary diversity and associated factors among children(6–23 months) in Gedeo zone, SNNPR, Ethiopia. FAO proposed the minimum dietary diversity for children consisting of 7 food groups and a dichotomous indicator to indicate minimum dietary diversity when consuming at least four food groups out of seven. However, in this study only 29.9%(199) of children were consumed a minimal dietary diversity before 24 h preceding the survey, this is slightly similar to the study conducted in Wolaita Sodo, Ethiopia (27.3%) [31]. The findings of this study were highly lower than the studies conducted in Kenya (45%), Zambia (37%), Indonesia (65%), Morocco (66%), Addis Abeba (59.9%) and Nepal of Asian country (30.4%) [32–34]. On the other hand, the result of this study was higher than previous studies conducted by WHO 2010 reports in Eretria (19%), Guinea (18%), India (12%) and Niger (5%) [33]. This discrepancy and similarity might be due to the variations of like geographical location, seasonal variability, sample size and other sociodemographic factors.

This study also indicated that the wealth index had a significant association with consuming of a diversified meal for children. Children living in the richest households were 10.4 times more likely to fed a diversified meal group than thus who were living in the poorest households [AOR4.390(2.300–8.380)]. This study is supported by the studies conducted in Nepal DHS, Dejen District, North West Ethiopia, Philippines and study conducted in 9 sub-Saharan African countries [35, 36]. This may indicate that family income has a direct association with household food security, since food consumption is believed to be heavily influenced by income of the household. This means that they may have ability to afford to have diversified foods as compared to children’s living in poorest households.

According to this study, educational status of mothers had statically significant association with dietary diversity of children. Children born from mothers who had formal education were 2.8 times more likely to consume the recommended minimum dietary diversity than those born from their mothers who can’t read and write [AOR 2.864(1.156–7.094)]. The finding of this study is in line with the study conducted in Ethiopia, Nepal, Indonesia, Bangladesh, five South Asian countries, Sri-Lanka and India [6, 37, 38]. Possible explanation for this might be due to the fact that those who had formal education would have an increased chance to get information regarding to nutritional requirement for their children’s and understand educational messages delivered through different media outlets. For this, as the educational level increases, the level feeding adequate dietary diversity for their children’s is expected to increase.

Furthermore, children born in families who had less than four children were 2.8 more likely to consume the recommended minimum dietary diversity than those who had four and above child [AOR 2.865(1.776–4.619))]. The finding of the present study is smellier with the finding of studies conducted at Kenya and Ethiopia [30, 39]. This could partly be explained by the fact that as the number of family member’s increases, the intrahousehold food distribution is affected and food may become more limited, which in turesprn would limit access to different food groups [30].

Moreover, Children who had between 9 and 11 months’ age group were 4.2 times more likely to consume the recommended minimum dietary diversity than those who had age between 6 and 8 months [AOR 4.237(1.743–10.295)]. The finding of the present study is smellier with the findings of other studies conducted at Dangila town, Gorche district in rural Ethiopia and 2016 Nepal DHS analysis [6, 37, 40, 41]. This might be due to infant’s mothers during 6–8 months who did not introduce solid and semisolid food; they are only introducing simply feeding milk along with breast milk. This is might be the perception of mothers, the poor ability of child’s intestine to digest solid, semisolid and soft foods. However, studies conducted at kemba woreda, southern Ethiopia, secondary Analysis of Ethiopian Demographic and Health Survey at 2011were controversial with this study [42, 43].

### Limitations of the study

The major limitation of this study was its cross-sectional design, which does not allow cause-effect relationships. It may not also accurately reflect the children’s past feeding experience, since it considers only 24-h feeding.

## Conclusion and recommendations

### Conclusions

Only one out of four children aged of 6–59 months received a minimum dietary diversity in the study area. In this study, number of families/children, household wealth index, educational status of mothers and age of children were significantly associated with the dietary diversity of children.

### Recommendation

Promoting the socioeconomic empowerment of women to increase the practice of dietary diversity for children. Facilitating the implementation of dietary diversity screening guide line so that the community will be aware of it during facility visit. Healthcare workers should counsel mothers on how they can increase food variety, especially mothers who have infants less than 1 year of age. There should be a series of trainings and support to the farmers in the area to promote production and consumption of diverse and nutritious foods to improve dietary diversity. Health facilities should be providing a sustained nutrition education program to mothers regarding dietary diversity and proper child feeding practices in collaboration with the respective religious leaders is highly recommended.

Further study has to be conducted in the area to determine adequacy of nutrient intake, on comparison of nutrient intake and nutritional status of children and the nutritional value of local available foods in the area.

## Data Availability

All data included in this manuscript can be accessed from the corresponding author upon request through the email address.

## Abbreviations

ANC: Antenatal care
CF: Complimentary feeding
DD: Dietary diversity
DHS: Demographic and Health Survey
EDHS: Ethiopian Demographic and Health Survey
FANTA: Food and Nutrition Technical Assistance project
FAO: Food and Agriculture Organization of the United Nations
GMP: Growth Monitoring program
HFIAS: Household Food Insecurity Access Scale
HH: Household
IYCF: Infant and young child feeding
MDD-C: Minimum dietary diversity of children
NGO: Non Governmental Organization
PNC: Postnatal care
PPS: Probability proportional to size
SNNPR: South Nations Nationalities and Peoples Region
SPSS: Statistical Package for the Social Sciences
SRS: Simple random sampling
UNICEF: United Nations Children’s (Emergency) Fund
USA: United States of America
USAID: United States Agency for International Development
VIF: Variance inflation factor
WHO: World Health Organization
